# Inexplicable or Simply Unexplained? The Management of Maize Seed in Mexico

**DOI:** 10.1371/journal.pone.0068320

**Published:** 2013-06-28

**Authors:** George A. Dyer, Alejandro López-Feldman

**Affiliations:** 1 Centro de Estudios Económicos, El Colegio de México; Abt Associates, Inc.; Instituto Nacional de Ecología y Cambio Climático, Mexico City, Mexico; 2 División de Economía, Centro de Investigación y Docencia Económicas, Mexico City, Mexico; United States Department of Agriculture, United States of America

## Abstract

Farmer management of plant germplasm pre-dates crop domestication, but humans’ role in crop evolution and diversity remains largely undocumented and often contested. Seemingly inexplicable practices observed throughout agricultural history, such as exchanging or replacing seed, continue to structure crop populations across the developing world. Seed management practices can be construed as events in the life history of crops and management data used to model crop demography, but this requires suitable quantitative data. As a prerequisite to addressing the causes and implications of maize seed management, we describe its patterns of variation across Mexico by drawing from the literature and new analysis. We find that rates of seed replacement, introduction and diffusion differ significantly across regions and altitudinal zones, but interactions among explanatory factors can obscure patterns of variation. The type, source, geographic origin and ownership of seed help explain observed rates. Yet, controlling for the characteristics of germplasm barely reduces interregional differences vastly exceeding variation across elevations. With few exceptions, monotonic altitudinal trends are absent. Causal relationships between management practices and the physical environment could determine farmers’ wellbeing and crop conservation in the face of climate change. Scarce and inconsistent data on management nevertheless could prevent an understanding of these relationships. Current conceptions on the management and dynamics of maize diversity are founded on a patchwork of observations in surprisingly few and dissimilar environments. Our estimates of management practices should shed light on differences in maize population dynamics across Mexico. Consistency with previous studies spanning over a decade suggests that common sets of forces are present within large areas, but causal associations remain unknown. The next step in explaining crop diversity should address variation in seed management across space and time simultaneously while identifying farmers’ values and motivations as underlying forces.

## Introduction

Adaptive radiation is perhaps the most obvious expression today of the evolution of maize, and one of its manifestations is a wide array of patterns associated with altitude [1–4]. Associations of crop diversity with altitude have been observed from the morphological to the molecular level. It has been known for over half a century that most maize races are restricted to certain altitudinal zones, while some sub-races are adapted to different elevations [1,5,6]. A strong correlation of isozyme variation with altitude (and to a lesser extent with latitude and longitude) was first noted 30 years ago [7,8]. Systematic analysis of maize collections later confirmed that the environment—as defined by altitude and geographic location—could explain the distribution of all known races of maize in Mexico [2]. The explanation for these patterns has been that, in Mexico, environmental conditions (e.g., temperature, precipitation, moisture and length of growing season) co-vary with altitude to a greater extent than either latitude or longitude [8,9]. Bretting et al. [10] vastly extended the breath of this explanation by suggesting that not only environmental conditions but also the distribution of human cultures, and hence agronomic practices, are determined by altitude. Alas, after more than two decades of social research, evidence supporting this hypothesis remains hard to find [11–15].

It has been suggested that the effect of farming practices on maize diversity and evolution is obscured by the strength of geographic and environmental factors, always defined in terms of latitude and altitude [15]. While acknowledging these factors’ “unambiguous” influence, Brush & Perales [15] seek to unravel farmers’ present-day contribution to maize evolution by analyzing the way in which the structure of human societies affects that of maize populations. Based on the most thorough survey of maize-farming communities to date, they conclude that in Chiapas, Mexico, the effect of social factors is evident only at the landscape level, while the environment contributes to maize diversity at greater spatial scales. Not surprisingly, their findings have been cited widely as evidence of the primacy of environmental factors compared to human’s relatively minor influence on maize diversity [5,6,9,15–18].

Disentangling the role of human and environmental factors in crop evolution could require a more appropriate conceptual framework than in use today, as well as better data [19,20]. In fact, the main impediment to identifying farmers’ continuing contribution to crop evolution could be the lack of systematic records on seed management practices—practices that at times have seemed inexplicable despite their presence throughout history [21]. With few exceptions, their role in the origin and spread of crops has been discussed only in passing [4,22]; yet, their part in assuring these crops’ future is once more a matter of public concern. In the face of climate change, the relationship between these practices and the physical environment with altitude could have vast implications for the wellbeing of farmers and the conservation of the genetic resources on which agriculture depends [6,23,24]. Recent reviews on crop seed management have discussed various conceptual issues but largely ignored the adequacy of quantitative data and its analysis [20,25–27]. In this paper we draw from the literature and new analysis to describe variation in maize seed management across Mexico. While attempting to identify the factors associated with this variation, we acknowledge that the volume of research and data available poses strict limits on any discussion of causality for various practices and regions.

## Methods and Data

In the last twenty years there has been an outpour of studies on the management of maize diversity in Mexico. In the southeast, arguably the most researched region, maize has been studied in Chiapas [11,14,15,28–32], Oaxaca [30,33–39], Veracruz [24,40], and Yucatán [23,41,42]. In Central Mexico, studies have been conducted in various localities in the state of Puebla [43–46] and across Puebla, Mexico and Morelos [12,24]. West-central Mexico also is well represented by studies in the states of Guanajuato [47–49], Jalisco [38,50], Michoacán [38,42,51] and Nayarit [52]. Other studies have taken place in Guatemala, part of maize’s primary center of diversity, and in its secondary center in Peru [53,54]. Unfortunately, few of these report consistent statistics on seed management or its consequences on crop demography. Management practices can be construed as events in the life history of crops and quantitative data used to estimate demographic parameters that can shed light on their population dynamics [55,56]; however, this requires that data be reported on a specific basis. While most practices can be reported in terms of either the farmers surveyed or the germplasm they grow, only the latter can be transformed into crop demographic parameters [55]. Another requisite is that the unit of reporting allows us to associate farmers’ decisions unequivocally with the populations they manage. This unit is the seed lot, i.e., the set of kernels of a specific type (e.g., shape, size or color) selected by a farmer and sown during a cropping cycle [50].

Primary data on 859 maize seed lots maintained by 606 households were used to estimate the rates at which seed lots are replaced (1 -p), diffused (q) (i.e., sold, exchanged or given away as gifts) and introduced (r) (i.e., transported) into a locality, and to identify the factors associated with these rates. The three rates can be construed as the probability that a seed lot will survive (p), reproduce (q) or migrate (r) [55]. Analyses were performed using log-linear and zero-inflated Poisson models following Dyer & Taylor [55]. Log-linear models compare proportions arranged into three-way tables [57]. G tests of goodness of fit were used to test the effect of individual explanatory factors; Freeman-Tuckey deviates were used to identify interactions between factors. These include geographic location—as defined by region and altitudinal zone—and the characteristics of the germplasm, such as the type, source, origin and ownership of seed (Table 1). The regions considered are the southeast, center, west-center and northern Mexico, i.e., the northeast and northwest are combined into a single region. Altitudinal zones are the lowlands, mid elevations and highlands.

**Table 1:** Seed management practices analyzed, and factors defining seed populations.

*Management practices (population parameters)*	
**Seed saving**	Sowing in the same farm seed harvested in a previous cycle (p)
Replacement	Not saving seed (1 -p)
Introduction	Transporting into a locality seed or grain to sow (r)
Diffusion	Selling, exchanging or giving away seed to sow (q)
***Geographic location***	
Region	Southeast, center, west-center, north
Altitude	Lowlands (0 - 1200 masl), mid elevations (1200–2000 masl), highlands (<2000 masl)
***Seed characteristics***	
Type	Landrace, improved variety
Source	Formal systems, informal seed systems, grain markets
Origin	Local, introduced
Ownership	Saved, newly acquired

A first model was used to test for differences across regions and altitudinal zones in the replacement of seed from all sources. The high replacement rate of germplasm obtained through formal channels is well established: seed obtained from institutional or commercial sources is replaced at higher rates than either seed acquired from other farmers or as grain [53,56]; but little is know about the pattern of variation for these last sources. Thus, a second model tested for differences in the replacement of seed from informal sources and grain, and their interactions across regions. Given the differences observed between these two groups, a separate model was used to repeat the first test for seed obtained from informal sources alone.

Replacement rates are known to depend on whether germplasm is acquired within or outside the locality where it is sown [53,55,56]; but the effect of the seed’s origin has not been disentangled from that of altitude or source. Thus, a final model tested for altitudinal differences in the replacement of local and introduced seed from informal sources at the national level. The same model was then tested independently for west-central and northern Mexico and for the southeast and central regions. The first three models were replicated to estimate rates of seed introduction and identify the factors associated with these rates. The first and third models were applied also to the analysis of seed diffusion. Finally, three-way tables were used to test for statistical differences in rates estimated using primary and secondary data whenever these were available.

**Figure 1:**
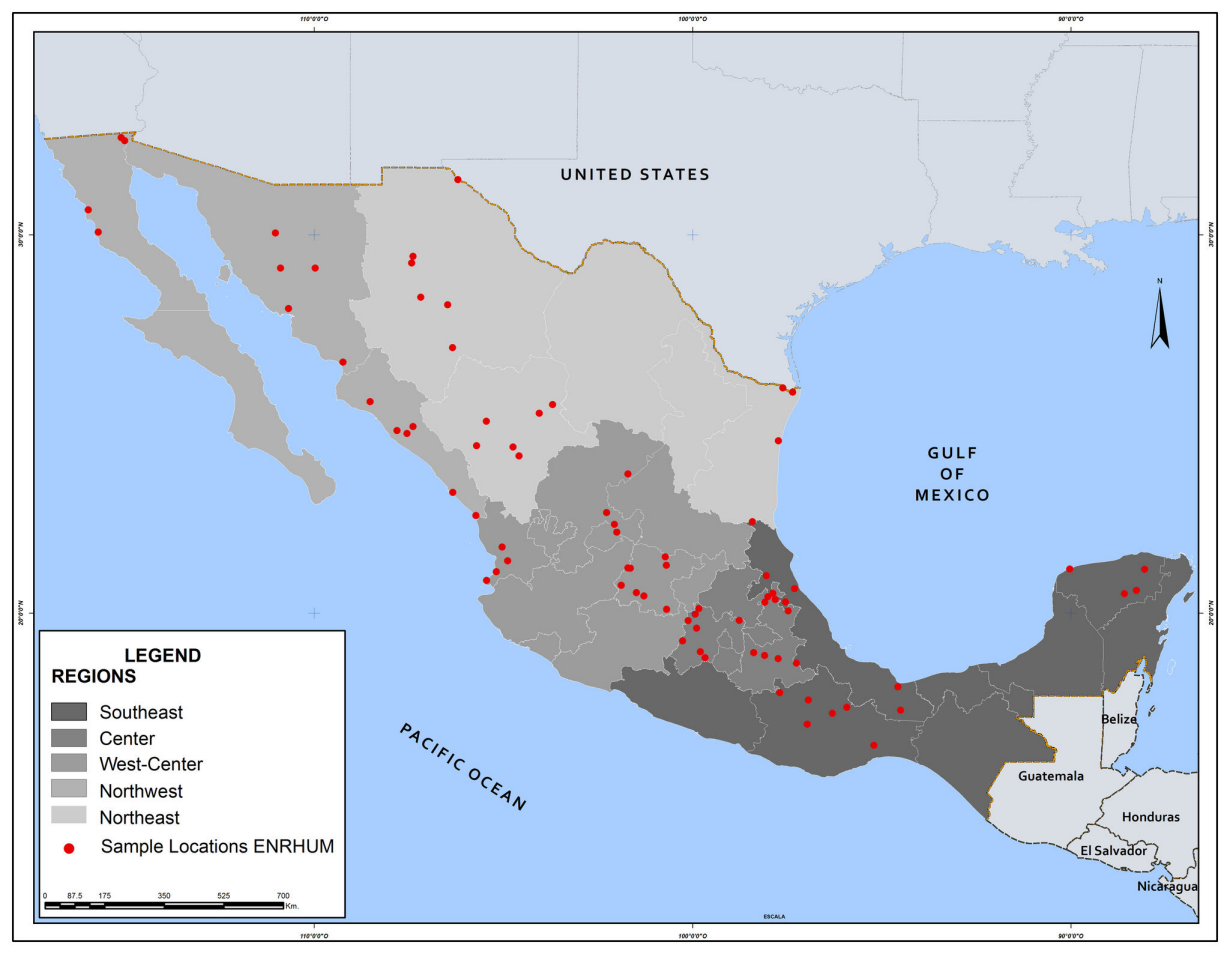
ENHRUM survey regions and locations. The Mexico Rural Household Survey (ENHRUM) is the source of primary data on the management of maize seed used in all analyses. ENHRUM is based on a stratified, three-stage, cluster sampling frame designed in collaboration with INEGI, the Mexican census bureau. A sample of states, localities and households (i.e., primary, secondary and elementary sample units, respectively), was selected through simple random sampling at every stage in each of the regions considered by INEGI. The sample is representative with 95% confidence of the rural population nationwide and in each region.

Primary data comes from the Mexico Rural Household Survey (ENHRUM), a collaborative effort of El Colegio de México and the University of California, Davis. (ENHRUM data are available at http://precesam.colmex.mx/ENHRUM.html.) The survey gathered detailed information on the activities and assets of the rural population, including data on every maize seed lot (i.e., every distinct seed type) managed by households in 2002, the time of the survey. ENHRUM is based on a stratified, three-stage, cluster sampling frame designed in collaboration with the Mexican census bureau (Instituto Nacional de Estadística, Geografía e Informática, INEGI). Within each of the five regions in which INEGI divides the country, a sample of states, localities and households (i.e., primary, secondary and elementary sample units, respectively) was selected through simple random sampling at every stage. The sample is representative with 95% confidence of the rural population nationwide and in each region (Figure 1). Since management practices are farmer decisions and management data are derived from a census of seed lots owned by surveyed households (i.e., not from a sample of seed lots), there are no sample design effects to consider besides those pertaining to the sampling of households. Although the degree of confidence for specific areas within regions—such as altitudinal zones (hereafter referred to as altitude-by-region environments)—is lower than 95%, test statistics reported below remain valid. The precision of estimates might be low nevertheless for environments where sample sizes are small.

In some cases, alternative estimates were made by pooling data from ENHRUM and various case studies reported in the literature. It should be noted nevertheless that the resulting estimates are not necessarily better or representative of any given environment. Few studies report the degree of confidence of their samples or the area of which they are statistically representative. Also, with few exceptions, studies do not report separate data for seed of various types or sources separately (e.g., landraces and improved varieties), and hence secondary data were used only in some analyses.

Separate estimates were made for the state of Chiapas, which was not covered by ENHRUM but was sampled extensively in a different study [15]. Given that these estimates differ significantly across elevations and ethnic groups, their validity rests on the assumption that the sample is representative of the state population, i.e., that sampling of mestizos and indigenous farmers reflects their relative abundance in every altitudinal zone.

## Results

### Seed replacement

A log-linear model reveals significant differences in replacement rates of seed across geographic regions (G = 137.1; P < 0.0001) and altitudinal zones (G = 26.9; P < 0.001). The lowest rate was observed in the southeast highlands, the highest in the northern lowlands (Table 2A), but no clear altitudinal or latitudinal trends were observed. At the national level, replacement rates were lowest in the highlands but highest at mid elevations. Across regions, rates were lowest in the southeast but highest in west-central Mexico. Freeman-Tukey deviates revealed important altitude-by-region interactions. Systematic decreases in replacement with altitude were observed only in southeastern Mexico. In northern Mexico, rates also were highest in the lowlands, but no significant differences between mid and high altitudes were observed. In central and west-central Mexico no significant differences were observed across altitudinal zones. Similarly, differences across regions were not independent of altitude. Regional differences observed above were not present at every elevation, i.e., in the lowlands, replacement was highest in the north and lowest in central Mexico.

**Table 2:** Rates of seed replacement in Mexico, by region, altitude and seed source^1^.

	All seed							Seed from informal sources			
	A. Altitude (N = 859)			B. Seed source (N = 673)				C. Altitude (N = 633)				
Region	Lowlands	Mid-altitudes	Highlands	Informal	Grain	Formal	Regional	Lowlands	Mid-altitudes	Highlands	Regional
Southeast	0.18	0.16	0.03	0.14	0.13	1.00	0.13	0.22	0.17	0.03	0.14
Central	0.14	0.15	0.20	0.16	0.43	0.67	0.18	0.15	0.07	0.18	0.16
West-central	0.65	0.70	0.65	0.56	0.80	0.95	0.67	0.50	0.55	0.57	0.56
North	0.75	0.45	0.29	0.33	0.90	1.00	0.55	0.44	0.28	0.50	0.33
Mexico	0.26	0.36	0.22	0.21	0.63	0.93	0.27	0.22	0.20	0.20	0.21
**G test for**											
region	G = 137.1*** (9 d.f.)			G = 72.7*** (6 d.f.)				G = 80.3*** (9 d.f.)			
altitude	G = 26.9*** (8 d.f.)							G = 23.6*** (8 d.f.)			
seed source				G = 17.3*** (4 d.f.)							

Significance at the 0.01 level is indicated by *** G tests exclude seed from formal seed systems.

^1^Expressed as a ratio, rates vary between 0 and 1 Replacement occurs when seed is not saved across cycles.

Replacement rates estimated using secondary data for 16 communities in central Mexico [12,24] are higher but not statistically different from those based on ENHRUM (G = 4.61; P = 0.20) (Table 3A). Pooling all data available for this region does not reveal significant differences across altitudinal zones (G = 4.71; P = 0.32).

**Table 3:** Rates of seed replacement in Mexico, by altitude, data source and seed origin.

	Seed from central region			Seed from informal sources, all regions		
	A. Data source^1^ (N = 746)			B. Seed origin^2^ (N = 661)		
**Altitude**	ENHRUM	Other	Pooled	Local	Introduced	Total
Lowlands	0.14	0.21	0.18	0.21	0.86	0.24
Mid-altitudes	0.15	0.18	0.17	0.20	0.42	0.25
Highlands	0.20	0.27	0.25	0.15	0.61	0.20
Total	0.18	0.25	0.22	0.18	0.55	0.22
**G test for**						
altitude	G = 4.71 (4 d.f.)			G = 8.53* (4 d.f.)		
data source	G = 4.61 (3 d.f.)					
seed origin				G = 46.9*** (3 d.f.)		

Significance at the 0.01 level is indicated by *** 0.10 level indicated by *

^1^ Sources are ENHRUM and Ref. [12,24].

^2^ Seed origin refers to the location of the immediate source of seed. Seed is “local” if obtained from sources within the locality; otherwise it is “introduced”.

A second model tested for differences in the replacement of seed obtained from informal sources and grain, and their interactions across regions (Table 2B). At the national level, seed obtained as grain was replaced at three times the rate of seed from informal sources (G = 17.3; P = 0.002). Similar differences were observed in every region but the southeast, where seed from both sources was replaced at the lowest rate of any region (1 - p = 0.14) (G = 72.7; P < 0.0001). Seed from grain was replaced at the highest rates in western Mexico (0.80) and particularly the north (0.90).

Clearly, rate estimates in Table 2A are influenced by differences in the replacement of seed from various sources and their relative abundance across regions, e.g., high replacement rates in west-central and northern Mexico could be due to the fact that germplasm acquired through formal channels, which is common there, often is replaced perfunctorily. A third model estimated exclusively for seed obtained from informal sources avoids this problem, revealing slightly different patterns from those described in Table 2A. Significant differences across geographic regions (G = 80.3; P < 0.0001) and altitudinal zones (G = 23.6; P = 0.003) persisted nevertheless (Table 2C). In this case, replacement was highest in western Mexico irrespectively of altitude. Replacement rates in northern Mexico were also higher than average. Rates were lowest in central Mexico, except at high elevations, where replacement was lowest in the southeast.

In every altitudinal zone, introduced seed from informal sources was replaced at higher rates than local seed (G = 46.9; P < 0.0001) (Table 3B). The effect of altitude on replacement was only marginally significant at the national level (G = 8.53; P = 0.07) because patterns differed across regions (data not shown). Nationwide, introduced seed was replaced at the highest rate in the lowlands and the lowest at mid elevations, but this occurred only in west-central and northern Mexico (G = 15.81; P = 0.003). In the southeast and central regions, introduced seed was replaced most at mid elevations, while local seed was replaced most in the lowlands (G = 9.45; P = 0.05). No differences in the replacement of local seed were observed in west-central and northern Mexico.

### Seed introduction

The rate of introduction differed significantly for seed from various sources (G = 131.9; P < 0.0001) and altitudinal zones (G = 10.6; P = 0.03). Seed obtained through informal channels was introduced at much lower rates than seed from other sources (Table 4A). No interactions between seed source and altitude were observed at the national level; seed form informal channels was introduced at lower rates in every altitude. Similarly, seed was introduced at the lowest rates in the lowlands and the highest rates at mid elevations irrespectively of source.

**Table 4:** Rates of seed introduction and diffusion in Mexico, by altitude, seed source and region^1^.

	Introduction rates							Diffusion rates^2^				
	A. Seed source (N = 744)		B. Region (N = 744)					C. Region (N = 739)				
Altitude	Informal	Other	Southeast	Central	West-central	North	Mexico	Southeast	Central	West-central	North	Mexico
Lowlands	0.03	0.50	0.03	0.01	0.27	0.50	0.09	0.19	0.30	0.38	0.04	0.23
Mid-altitudes	0.09	0.74	0.06	0.07	0.58	0.22	0.22	0.34	0.10	0.21	0.01	0.21
Highlands	0.06	0.56	0.02	0.07	0.25	0.03	0.09	0.30	0.18	0.24	0.14	0.22
Total	0.06	0.61	0.03	0.06	0.37	0.31	0.12	0.25	0.19	0.25	0.04	0.22
**G test for**												
altitude	G = 10.6** (4 d.f.)		G = 26.4*** (8 d.f.)					G = 10.6** (4 d.f.)				
source	G = 131.9*** (3 d.f.)											
region			G = 111.8*** (9 d.f.)					G = 34.4*** (6 d.f.)				

Significance at the 0.01 level is indicated by *** 0.05 level indicated by ** G-tests exclude seed from formal seed systems.

^1^ Expressed as a ratio, rates vary between 0 and 1 Introduction implies that seed is transported into the locality; diffusion entails the exchange of seed among farmers.

^2^ G tests lump mid and high altitudes.

At the regional level, however, this was observed only in west-central and southeastern Mexico (G = 26.4; P < 0.001) (Table 4B). In central Mexico, rates were the same at mid and high elevations, while in northern Mexico they were highest at low elevations. The rate of introduction also differed significantly across regions (G = 111.8; P < 0.0001): rates were highest in west-central Mexico and lowest in the southeast, but again, this was not true in every altitudinal zone. In the lowlands, rates were highest in the north and lowest in central Mexico. These patterns were not affected markedly by the varying abundance of seed from formal sources in each zone and region. When seed from informal sources was analyzed separately, the patterns remained basically unaltered although the rates themselves were halved (data not shown). Introduction rates estimated using secondary data for three altitude-by-region environments [24] were not statistically different from those based on ENHRUM data, but pooling all data revealed altitudinal differences in central Mexico in greater detail than before (Table 5A).

**Table 5:** Rates of seed introduction and diffusion in Mexico, by altitude and data source.

	Introduction rates					Diffusion rates			
	A. Data source^1^					B. Data source^1^			
Altitude	ENHRUM	Other	Pooled	Chi^2^		ENHRUM	Other	Pooled	Chi^2^
SE lowlands	0.03	0.01	0.02	0.41		0.19	0.25	0.23	1.21
C mid-altitudes	0.07	0.00	0.03	2.60		0.10	0.29	0.21	4.3**
C highlands	0.07	0.07	0.07	>0.01		0.18	0.18	0.18	0.02

Significance at the 0.05 level is indicated by ** Chi^2^ degrees of freedom = 1.

^1^Sources are ENHRUM and Ref. [24].

### Seed diffusion

Altitude had significant effects on the diffusion of seed in every region, but only marginal differences were observed at the national level (G = 10.6; P = 0.05). Various altitude-by-region interactions were present. In central and west-central Mexico, seed was diffused most in the lowlands; in the southeast, it was diffused most at mid and high elevations (Table 4C). Conversely, in the highlands and mid elevations, seed was diffused at the highest rates in the southeast, while in the lowlands it was diffused most in west-central Mexico (G = 34.4; P < 0.001). The same pattern and similar rates were observed for seed from informal sources (G = 15.7; P = 0.02) (data not shown).

Diffusion rates estimated using secondary data and ENHRUM were not statistically different in the central highlands (Chi^2^ = 0.02; P = 0.96) and southeastern lowlands (Chi^2^ = 1.21; P = 0.27), but differences were observed at mid elevations in central Mexico (Chi^2^ = 4.37; P = 0.04) (Table 5B). It was not possible to test whether differences were due to the unusually low frequency of improved varieties recorded by the previous study since data for different types of germplasm was not reported separately [24].

Diffusion rates reflect the probability that a seed lot is diffused in a given year but not the number of times it is diffused (or diffusion events). A zero-inflated-Poisson regression model reveals that the number of diffusion events (conditional on the seed lot being diffused) is higher in the highlands than at other elevations (z = 1.87; P = 0.07). It is lower for saved and local seed and for landraces in general, but it increases with age. A Vuong test supports use of this model over a single-stage Poisson model (P < 0.001).

## Discussion

In many areas in Mexico, on-farm maize diversity is structured into numerous populations evolving sympatrically under multiple human and environmental forces [23,36]. Brush and Perales [15] describe these populations in the state of Chiapas. Average statistics for the state, such as the rate of seed replacement (1 - *p* = 0.11), seem of little significance given the wide variation across altitudes. In Chiapas, the age, source and replacement rates of seed all vary monotonically with altitude. In lowland localities, for instance, acquaintances outside the family are the most important source of seed, while family sources become increasingly important at higher elevations. Only 3% of seed lots maintained by indigenous highland farmers are replaced annually (i.e., 1 - *p* = 0.03); the average age of a seed lot is 28.5 years. Unsurprisingly, a large percentage (83.9%) of this germplasm has been transmitted from parents to their kin. In contrast, mestizo farmers in the Chiapas lowlands replace one fifth of their seed every year (i.e., 1 - *p* = 0.20) and the average age of this germplasm is 10 years. The authors find also that altitude’s association with seed management is independent of ethnicity, the other determinant of seed dynamics examined, i.e., they observe no significant interaction between the two factors. As mentioned, these two findings are interpreted widely as evidence of the dominance of environmental factors over human influence. Whether such interpretation is valid or not, the generality of the observations on which it is based is questionable.

A correlation between seed replacement and altitude has been observed before outside Chiapas [12,53]; however, a recent nationwide study found only weak evidence of an association [56]. The reason, our results show, is that the distinctive association reported previously is present throughout the southeast but not in other regions (Table 2A). That is, a consistent, monotonic pattern of variation in replacement with altitude occurs only in the southeast. Seed replacement varies to a much greater extent across regions than across elevations, e.g., rates in west-central Mexico are five times those found in the southeast (Table 2A). Furthermore, interregional variation is not reduced markedly after controlling seed characteristics associated with replacement, e.g., seed source (Table 2C). Accounting for both location and seed characteristics is crucial nevertheless because frequent interactions among factors (e.g., altitude-by-region and altitude-by-source interactions) can obscure the patterns of variation. For instance, the aforementioned study found few differences in replacement across altitudinal zones but a consistent pattern of variation within each zone, where seed from formal sources was replaced at higher rates than seed from grain, and the latter at higher rates than seed from informal sources [56]. Our results confirm that replacement rates depend on the source and origin of seed as well as altitude, but variation within individual regions rarely conforms to the general pattern just described (Table 2A and B). Variation is distributed in unique patterns for each practice within each environment. These patterns are undoubtedly the outcome of the multiple forces shaping maize populations in that particular environment; but patterns do not necessarily reveal underlying causes. In what follows we discuss in detail regional variation in various practices; nationwide patterns have been discussed previously [55,56].

### Seed Replacement

#### Southeast Mexico

The rate of seed replacement estimated for southeast Mexico based on ENHRUM data is strikingly close to the independent estimate for Chiapas (0.13 vs. 0.11, respectively) [15]. Chiapas is located within the region but was not part of ENHRUM. Throughout the southeast, as in Chiapas, seed replacement rates decrease consistently with altitude. The region’s highlands exhibit the lowest replacement rate of any altitude-by-region environment in Mexico (Table 2A), which also is very close to the estimate for highland Chiapas (0.03 and 0.04, respectively). Some of the variation observed across altitudinal zones can be attributed to differences in the relative abundance of various types and sources of germplasm. In the southeast, as elsewhere in Mexico, improved germplasm acquired from formal sources is replaced at much higher rates than landraces [55,56]. The near absence of seed from formal sources in the southeast, particularly in the highlands, thus helps explain the low replacement rates in this environment. However, other germplasm also is replaced less in the southeast highlands than elsewhere in Mexico (Table 2C).

As stated above, the low rates of replacement observed in highland Chiapas have been attributed to the combination of two independent factors: altitude and ethnicity [15]. Indeed, ethnicity seems to explain considerable variation in replacement rates at every elevation in Chiapas: indigenous farmers consistently replace seed less often than mestizos. At mid elevations, for instance, seed replacement rates are almost twice as high among mestizos as among indigenous farmers (i.e., 0.07 vs. 0.04). Ethnicity and altitude could have similar roles in seed replacement throughout the southeast, but additional sources of variation clearly are at play within the region. For instance, average replacement rates at southeastern mid elevations vary considerably across states. An independent study in the strongly indigenous central valleys of Oaxaca suggests a rate twice that estimated for similar elevations in Chiapas (i.e., 0.10 vs. 0.05) [15,33]. This observation is supported by ENHRUM data for Oaxaca’s mid elevations, where average rates are higher than for both indigenous and mestizo farmers in Chiapas, and hence not easily explained by ethnicity. Similarly, improved varieties and their advanced generations are less common in Oaxaca than in Chiapas [15,33], so observed differences across these states are the opposite of what we might expect based on the relative abundance of various types of germplasm. These differences could be tied to temperature and precipitation, as mid elevations in Oaxaca are noticeably dry.

While not all sources of variation within this environment have been identified, the region’s mid elevations clearly experience distinct rates of seed replacement. According to ENHRUM data, the average replacement rate at mid elevations (0.16) differs significantly from rates in the highlands and lowlands (Table 2A). Although the rate decreases when data from other studies in this altitude-by-region environment are considered (0.08), differences with other altitudinal zones persists.

As to the region’s lowlands, according to ENHRUM data the average rate of seed replacement in this environment is exactly the same as that estimated for lowland Chiapas (i.e., 0.18) [15]. As at higher elevations, some variation in the lowlands might be associated with ethnicity. In fact, rate differences between indigenous farmers and mestizos (0.12 vs. 0.20, respectively) are greater in lowland Chiapas than at other elevations [15]. Additional variation could be attributed to the distribution of improved germplasm, which is considerably more common in the lowlands than at higher elevations but also highly aggregated. An independent study in La Frailesca—a prime agricultural and largely mestizo area in Chiapas—reported a considerably high replacement rate (0.57) [31]. This rate is well above the average rate for lowland Chiapas, which includes regions like the Usumacinta river basin where maize landraces are predominant. The relatively low rates in the southeast lowlands, compared to other regions, also reflect the abundance of advanced generations of improved germplasm in this environment [15,55].

#### Central Mexico

An association between altitude and seed management was first observed a decade ago in central Mexico [12]. It was reported then that replacement rates increased consistently along a transect running south from the Valley of Mexico’s highlands to the mid elevations of the Valley of Cuautla, where rates were noticeably high (0.37) due to the preponderance of improved germplasm. Findings in Chiapas seemed to confirm the generality of this pattern [15], which as noted above, is widespread in the southeast; but surprisingly, the pattern is not the norm in central Mexico. In fact, seed replacement in central Mexico differs considerably from that observed in the southeast.

In general, rates of seed replacement are almost 40% higher in central Mexico (1 - *p* = 0.18) than in the southeast (Table 2A); and in contrast to the latter region, rates vary slightly across altitudinal zones. Indeed, neither the high rates observed in Cuautla nor the low rates in the Valley of Mexico are representative of the region. Arguably, the main altitudinal gradient in central Mexico—sampled extensively in a recent study [24]—runs east from the highlands in the Valley of Toluca to the lowlands in the Gulf of Mexico. According to that study, replacement rates actually drop from the highlands (0.27) to the mid elevations (0.18), where little improved germplasm was found. ENHRUM data suggests a smaller albeit not significant decrease from high to mid elevations (0.20 and 0.15, respectively), while the rate in the lowlands (0.14) is not statistically different either (Table 2A). Lack of differences is confirmed when all available data for the region are considered (Table 3A, but also see below).

Differences between central and southeast Mexico are partly explained by a greater abundance of germplasm from formal sources in the former region and differences in its distribution across altitudinal zones. In contrast to the southeast, improved varieties are common in the central highlands and particularly the mid elevations while nearly absent in the lowlands. Differences in the management of seed acquired as grain (Table 2C), which in central Mexico (in contrast to the southeast) is replaced more than seed from informal sources, also help explain these patterns. Seed from informal sources is replaced at almost the same rate in central and southeast Mexico (0.16 vs. 0.14, respectively), but rates still differ considerably across regions when compared at similar elevations (Table 1 C). In the highlands, for instance, seed from informal sources is replaced at rates six times higher in central Mexico than in the southeast (0.18 vs. 0.03, respectively), while the opposite is true at mid elevations (0.07 vs. 0.17) (Table 2C). This pattern persists after pooling all data available for the region, and its causes remains unexplained.

Altitudinal differences become evident in central Mexico only when germplasm from informal sources is considered separately. Replacement rates for this seed are lower in the mid elevations than in either the highlands or lowlands (Table 2C). In fact, rates in the central mid elevations (0.07) are the lowest nationwide after the southeast highlands. In the former environment, nevertheless, low replacement rates cannot be attributed to either altitude or ethnicity (as in the southeast highlands) given that indigenous farmers are more numerous in both the central highlands and lowlands than at mid elevations (unpublished data).

Differences in replacement across regions are smallest at low elevations. When all data available area pooled, the rates for the central and southeast lowlands are the same (i.e., 0.18) (Tables 2A & 3A). This is surprising nevertheless considering the absence of improved varieties in the former environment. Seed from informal sources is replaced at slightly lower rates in the central lowlands than in the southeast (0.19 and 0.22, respectively; pooled data, not shown); yet, this rate too seems high given the strong indigenous presence in the central lowlands. An independent study in the Sierra Norte de Puebla, located within this environment, reported a relatively high rate (0.21) due to landless farmers renting land—and then borrowing seed—to grow maize [58]. Replacement also varied across maize types: rates were 0.24 for white maize, 0.20 for yellow maize and 0.13 for blue maize [58]. In contrast, no differences between color types were found in Jacaltenango, Guatemala, where replacement rates are even higher despite a strong indigenous presence [53]. The implication is that, notwithstanding common patterns, local replacement rates can be influenced by a unique assortment of factors. The dynamics of land use or the relative abundance of specific varieties, for instance, may play a role in some localities but not in others.

#### West-central and north Mexico

A recent review of the literature describes seed replacement as fairly conservative across the developing world [20], but the practice is extremely common in Mexico outside the central and southeast regions. The high rates in west-central and north Mexico are not explained by the predominance of improved germplasm, for landraces too are replaced at much higher rates than elsewhere [55]. In fact, higher replacement rates than in other regions are observed whether seed is obtained from formal or informal sources or as grain (Table 2B). This does not prevent differences in replacement based on the source of seed. In both west-central and north Mexico, grain is replaced at much higher rates than seed from informal sources, while seed from formal sources is replaced almost pervasively (Table 2B). Seed in general is replaced at a higher rate in west-central Mexico than in the north. Differences are absent at low elevations, but they become evident as replacement rates decrease in the North’s mid and high elevations. In this case, differences are due entirely to the replacement of seed from informal sources (Table 2C) since other seed is replaced at the same high rate.

West-central Mexico is the only region where seed is replaced at the same rate across altitudinal zones whether it is from informal sources or not, and notwithstanding differences in ethnic composition. The strongly indigenous Cuzalapa watershed, the setting of the seminal study on seed management [50], is located in the west-central lowlands. The high rates of replacement observed in that study (0.47) are characteristic of the entire lowlands, where the estimated rate is only slightly higher (0.50) (Table 2C). Yet, this rate is not significantly different from the rest of the region, where mestizo farmers predominate.

#### Seed source and origin

As stated above, previous studies have found that the rate of seed replacement is associated with both the origin and source of germplasm. In general, seed acquired as grain is replaced at 3 times the rate of that obtained through informal channels [56]. Similar patterns are present at different elevations and in every region but the southeast (Table 2B). Likewise, at the national level, introduced seed is replaced at more than thrice the rate of local seed, particularly at low elevations, where replacement rates are considerably high (0.81) [55,56]. However, these observations do not preclude the possibility that the effect attributed to the origin of seed is in fact due to its source or vice versa. This could be the case because seed from formal sources, which is replaced at very high rates and is most common in the lowlands, is non-local (see below). Alternatively, farmers in the southeast may not treat grain differently because they recognize it as local germplasm. While this remains an open question, our results show that the significance of origin is not due to the perfunctory replacement of improved germplasm. Origin has an effect also on the replacement of germplasm from informal sources: introduced seed (i.e., seed obtained from farmers outside the locality) is replaced three times more than local seed (Table 3B).

### Seed introduction

The regular movement of seed across localities documented in the valley of Cuzalapa [50] has led scholars to conclude that maize populations are essentially open systems [13,36]. Introduction rates reported for Cuzalapa (r = 0.11) actually underestimate the frequency of this practice there because seed acquired in different localities within the valley was considered local. According to ENHRUM data, the rate of introduction (across individual localities) in the west-central lowlands is considerably higher (0.26) than that reported for Cuzalapa. Moreover, this rate is less than half that observed in the region’s mid elevations (0.58). Most authors take for granted that the acquisition of seed from distant sources, such as that observed in Cuzalapa, is common throughout Mexico [19,36]. Yet, much of what is reported about seed exchange is anecdotal, and there are surprisingly few systematic data available. Reassuringly, the only commensurable data known to us [24] suggests that ENHRUM is a reliable source of statistics at a relatively fine scale, i.e., the altitude-by-region environment. And the data shows that the frequency of this practice is extremely variable across Mexico (Table 4B). In contrast to Cuzalapa, seed introduction can be remarkably rare or even absent in some environments, such as the central lowlands and mid elevations (0.01 and 0.03, respectively). Significantly, it is these extremely low rates that are confirmed by the aforementioned study (Table 5A) [24].

Our analysis also highlights the lack of independence among the factors that determine introduction rates, due in this case to altitude-by-region interactions. Namely, the pattern of variation across altitudinal zones that is highly significant at the national level—i.e., introduction rates are most common at mid elevations—occurs only in southeastern and west-central Mexico, while other regions exhibit contrasting patterns: in the north, seed is introduced most in the lowlands, in central Mexico it is introduced most in the highlands. It would be tempting to explain this pattern, most clearly in northern Mexico, through reference to the preponderance of improved germplasm, which is introduced at the highest rate there and is most common at low elevations [55]; but this would not be justified. Notwithstanding considerable differences in the rates of introduction and relative abundance of seed from various sources, these bear no weight on the regional and altitudinal patterns described above. Essentially the same contrasting patterns are present for seed acquired through informal systems as for seed in general: seed is introduced most at mid elevations in west-central Mexico (0.27) and the northern lowlands (0.33), while rates in various environments in central and southeastern Mexico are an order of magnitude lower (data not shown).

Improved varieties as much as landraces can be acquired locally or outside the locality, which does not imply that both types of germplasm are introduced at equal rates; differences between landraces and improved varieties are evident in every region [55]. Although seed source and type are highly correlated, it is more meaningful to explain rates of introduction based on the seed’s source than on its type. It is its source that is directly associated with the seed’s origin, for clearly, germplasm obtained through formal channels must be considered non-local even if seed companies (or research institutions) distribute it directly in the locality [35,50]; i.e., seed obtained from formal sources is necessarily non-local [53].

Analyses based on the source of seed (rather than its type) also can control for a third possibility, namely seed obtained as grain, which often is managed indistinctly whether it is a landrace or an improved variety [56]. That grain seed is a distinct and meaningful category is shown by its price, which can be considerably lower than that for seed of the same variety [33,34,52]. Grain can be acquired locally or outside the locality, and its provenance can have important implications for the crop’s genetic makeup [56]. However, rates of introduction can be misguiding because the origin of grain cannot be ascertained in the same way as that of seed proper. In fact, one of the peculiarities of this source of germplasm is the lack of information that accompanies it.

In sum, the sources of seed can be associated confidently with distinct rates of introduction (Table 4A), which also differ considerably across regions and altitudes. However, in contrast to the seed’s source, neither region nor altitude has a systematic effect on introduction rates (Table 4B). As with seed replacement, additional sources of variation are most likely at play.

### Seed diffusion

Despite the importance conferred to seed exchange by biologists and social scientists [35,36], published data suitable to estimate diffusion rates is surprisingly scarce. Estimates based on the few data available coincide broadly with those reported here (Table 5B), corroborating ENHRUM’s reliability as a source of statistics. Studies based on ENRHUM have found that factors affecting seed replacement and introduction decisively (e.g., the type, source and origin of germplasm) have at best a weak influence on diffusion rates [55,56]. At the same time, there have been indications that the absence of statistically significant effects is due to strong interactions between these factors. Region-by-origin interactions, for instance, obscure the effect of the germplasm’s origin because introduced seed is diffused at higher rates than local seed in the southeast but at lower rates in west-central Mexico. Origin-by-ownership interactions are equally important. Evidence suggests that saved seed acquired locally might diffuse more than expected while new introduced seed diffuses less [55]. Altitude-by-origin interactions similarly come into play: local seed is diffused at higher rates than introduced seed but only at low and mid elevations [56]. Controlling for altitude also reveals that saved seed in general is diffused at higher rates than newly acquired seed, but no altitudinal effects on diffusion rates had been found to date [56]. Such effects become evident nevertheless within regions (Table 4C). In central and west-central Mexico, seed is diffused most in the lowlands; in the southeast, it is diffused most at mid and high elevations.

Finally, analysis of the number of times a given seed lot is diffused (i.e., the number of diffusion events per seed lot) has shown that this variable is associated with the age, ownership and origin of seed [55]. Our results show that the number of diffusion events also differs significantly across altitudinal zones. Controlling for altitude reveals additionally that while the probability of diffusion is higher for landraces than for improved varieties, the opposite is true of the number of diffusion events.

### Concluding remarks

Seed management (or crop diversity) is “explained” when its variation is controlled statistically, revealing an underlying pattern [15]. We have described management practices for different maize populations across Mexico and in the process explained them in this narrow sense. The management of maize seed exhibits recognizable patterns in various regions and altitudinal zones: rates of seed replacement, introduction and diffusion differ significantly across these environments. Estimates are relevant to the extent that they reflect the multiple forces shaping maize populations in a particular environment; they also constitute the crop’s demographic parameters. That they are consistent with observations gathered from various independent studies suggests that a common set of forces is present within each environment. Our analysis also shows that the management of maize germplasm is influenced decisively by the nature of seed, including its type, source, origin and ownership. Controlling for the characteristics of seed also uncovers significant differences across altitudinal zones; yet it fails to reveal a consistent association with altitude. The altitudinal patterns that emerge after controlling seed characteristics continue to differ markedly across regions.

These discrepancies should remind us that altitude cannot provide a definitive explanation of crop management (or diversity) but at best a proxy for missing explanatory factors, including but not limited to climate. More importantly, discrepancies also suggest that critical causal relationships underlying crop management (and diversity) remain unidentified. Individual factors discussed here offer only conditional explanations; their influence is rarely independent of other factors. For instance, replacement is not consistently lowest in the highlands or unfailingly higher for improved varieties than for landraces; rate differences between two types of seed can be reversed depending on other characteristics. The complete set of factors could be considered a definitive explanation only to the extent that it accounted for all major sources of variation and their causal relationships—that is, if it revealed the underlying processes—which is clearly not the case here.

Our explanation must be considered provisional for it neglects crop management’s proximate causes—farmers’ decision-making process—and their ultimate driving force, i.e., value. It assumes that all farmers respond identically to uniform conditions within each (altitude-by-region) environment. This happens because, with the exception of ethnicity, variables associated with farmers have not been included in the equation thus far. Although climate change could have an effect in the long run [6,24], our analysis implicitly assumes that management practices are unvarying through time, since ethnicity, environmental conditions and the properties of seed are given. Our perspective’s major weakness is thus that it constitutes a snapshot—it leaves unexplained the heterogeneity of farmers’ decisions and their changes from year to year, which has myriad implications for crop diversity [20,55].

Countless contingent factors and transient circumstances affect the management of crops and hence their diversity [20]. Some of them, such as natural disturbances, might be momentary or episodic while others constitute trends or cycles like those observed by agricultural prices and policies [32,59]. An in-depth explanation of crop management thus entails understanding how farmers respond to these events as well as the attitudes and perceptions that motivate their responses [44]. These may seem inexplicable at times, or entirely irrational, which indeed might be the case. A definitive explanation nevertheless requires recognizing that farming decisions are seldom taken in isolation; that a given practice usually depends on many others taken by either the same farmers, their neighbors or more distant members of society; or that crop management is not only caused by environmental conditions but the cause of those conditions. Such a system’s perspective to crop evolution is necessarily part of a long-term research agenda.

## References

[1] Wellhausen EJ, Roberts LM, Hernandez E, Mangelsdorf PC (1952) Races of Maize in Mexico, Their Origin, Characteristics, and Distribution. Cambridge, MA: The Bussey Institution, Harvard University. 223pp.

[2] Sanchez JJ, Goodman MM (1992) Relationships among the Mexican races of maize. Econ Bot 46: 72-85. doi:10.1007/BF02985256.

[3] Matsuoka Y, Vigouroux Y, Goodman MM, Sanchez GJ, Buckler E et al. (2002) A single domestication for maize shown by multilocus microsatellite genotyping. Proc Natl Acad Sci U S A 99: 6080-6084. doi:10.1073/pnas.052125199. PubMed: 11983901. 11983901 10.1073/pnas.052125199 PMC122905

[4] Vigouroux Y, Glaubitz JC, Matsuoka Y, Goodman MM, Sanchez J et al. (2008) Population structure and genetic diversity of New World maize races assessed by DNA microsatellites. Am J Bot 95: 1240–1253. doi:10.3732/ajb.0800097. PubMed: 21632329. 21632329 10.3732/ajb.0800097

[5] Ruiz Corral JA, Duran Puga N, Sanchez Gonzalez JJ, Ron Parra J, Gonzalez Eguiarte DR et al. (2008) Climatic adaptation and ecological descriptors of 42 Mexican maize races. Crop Sci 48: 1502-1512. doi:10.2135/cropsci2007.09.0518.

[6] Mercer KL, Perales HR (2010) Evolutionary response of landraces to climate change in centeres of crop diversity. Evol Appl 3: 1-15. doi:10.1111/j.1752-4571.2009.00093.x. 25567941 10.1111/j.1752-4571.2010.00137.x PMC3352508

[7] Goodman MM, Stuber CW (1983) Races of maize VI. Isozyme variation among races of maize in Bolivia. Maydica 28: 169-187.

[8] Doebley J, Goodman MM, Stuber CW (1985) Isozyme variation in the races of maize from mexico. Am J Bot 72: 629-639. doi:10.2307/2443674.

[9] Mercer K, Martinez-Vasquez A, Perales HR (2008) Asymmetrical local adaptation of maize landraces along an altitudinal gradient. Evol Appl 1: 489-500. doi:10.1111/j.1752-4571.2008.00038.x. 25567730 10.1111/j.1752-4571.2008.00038.x PMC3352380

[10] Bretting PK, Goodman MM, Stuber CW (1990) Isozymatic variation in Guatemalan races of maize. Am J Bot 77: 211-225. doi:10.2307/2444643. 10.1002/j.1537-2197.1990.tb13547.x 30139070

[11] Brush SB, Bellon M (1988) Corrales, Schmidt E. Agric Dev Maize Divers Méx Hum Ecol 16: 307-328.

[12] Perales H, Brush SB, Qualset CO (2003) Dynamic management of maize landraces in central Mexico. Econ Bot 57: 21-34. doi:10.1663/0013-0001(2003)057[0021:DMOMLI]2.0.CO;2.

[13] Perales RH, Benz BF, Brush SB (2005) Maize diversity and ethnolinguistic diversity in Chiapas, Mexico. Proc Natl Acad Sci U S A 102: 949-954. doi:10.1073/pnas.0408701102. PubMed: 15640353. 15640353 10.1073/pnas.0408701102 PMC545571

[14] Benz B, Perales H, Brush S (2007) Tzeltal and Tzotzil farmer knowledge and maize diversity in Chiapas, Mexico. Curr Anthropol 48: 289-300. doi:10.1086/512986.

[15] Brush SB, Perales HR (2007) A maize landscape: ethnicity and agrobiodiversity in Chiapas Mexico. Agric Ecosyst Environ 121: 211-221. doi:10.1016/j.agee.2006.12.018.

[16] Hart JP (2008) Evolving the three sisters: The changing histories of maize, bean, and squash in New York and the Greater Northeast. Chapter 7. In: Hart JP New York: Current Northeast Paleoethnobotany II State Museum Bulletin 512. pp. 87-99

[17] Perales H, Rivera Aguirre (2008) Biodiversidad humanizada. In: Capital natural de México, vol. I: Conocimiento actual de la biodiversidad. México: Conabio. pp. 565-603

[18] Astier M, Barrera-Bassols N, Odenthal J, Ramirez MI, Orozco Q et al. (2010) Participatory identification and mapping of maize diversity in the Pátzcuaro-Zirahuen Basins, Michoacan, Mexico. J Maps. doi:10.4113/jom.2010.1101.

[19] Brush SB (2004) Farmers’ bounty: Locating crop diversity in the contemporary world. New Haven, CT: Yale University Press. 327pp.

[20] Hodgkin T, Rana R, Tuxill J, Didier B, Subedi A et al. (2007) Seed systems and crop genetic diversity in agroecosystems. In: Jarvis DI Padoch C Cooper D Managing Biodiversity in Agricultural Ecosystems. New York: Columbia University Press pp. 77–116.

[21] Zeven AC (1999) The traditional inexplicable replacement of seed and seed ware of landraces and cultivars: A review. Euphytica 110: 181-191. doi:10.1023/A:1003701529155.

[22] Hillman GC, Davies MS (1990) Domestication rates in wild-type wheats and barley under primitive cultivation. Biol J Linn Soc 39: 39–78. doi:10.1111/j.1095-8312.1990.tb01611.x.

[23] Jarvis DI, Brown AHD, Pham HC, Collado-Panduro L, Latournerie-Moreno L et al. (2008) A global perspective of the richness and evenness of traditional crop-variety diversity maintained by farming communities. Proc Natl Acad Sci U S A 105: 5326-5331. doi:10.1073/pnas.0800607105. PubMed: 18362337. 18362337 10.1073/pnas.0800607105 PMC2291090

[24] Bellon MR, Hodson D, Hellin J (2011) Assessing the vulnerability of traditional maize seed systems in Mexico to climate change. Proc Natl Acad Sci U S A 108: 13432-13437. doi:10.1073/pnas.1103373108. PubMed: 21825131. 21825131 10.1073/pnas.1103373108 PMC3158171

[25] Thomas M, Dawson JC, Goldringer I, Bonneuil C (2011) Seed exchanges, a key to analyze crop diversity dynamics in farmer-led on-farm conservation. Genet Resour Crop Evol 58: 321-338. doi:10.1007/s10722-011-9662-0.

[26] De Boef WS, Dempewolf H, Byakweli JM, Engels JMM (2012) Integrating genetic resource conservation and sustainable development into strategies to increase the robustness of seed systems. J Sust Agric 34: 504-531.

[27] Pautasso M, Aistara G, Barnaud A, Caillon S, Clouvel P et al. (2012) Seed exchange networks for agrobioversity conservation: a review. Agron Sustain Dev. doi:10.1007/s13593-012-0089-6.

[28] Bellon MR, Brush SB (1994) Keepers of maize in Chiapas, Mexico. Econ Bot 48: 196-209. doi:10.1007/BF02908218.

[29] Bellon MR, Risopoulos J (2001) Small-scale farmers expand the benefits of improved maize germplasm: A case study from Chiapas, Mexico. World Dev 29: 799-811. doi:10.1016/S0305-750X(01)00013-4.

[30] Bellon MR, Adato M, Becerril J, Mindek D (2005) Poor farmers’ perceived benefits from different types of maize germplasm: the case of creolization in lowland tropical Mexico. World Dev 34: 113-129.

[31] Keleman A, Hellin J, Bellon MR (2009) Maize diversity, rural development policy, and farmers’ practices: lessons from Chiapas, Mexico. Geogr J 175: 52-70. doi:10.1111/j.1475-4959.2008.00314.x.

[32] Bellon MR, Hellin J (2011) Planting hybrids, keeping landraces: Agricultural modernization and tradition among small-scale maize farmers in Chiapas, Mexico. World Dev 39: 1434-1443. doi:10.1016/j.worlddev.2010.12.010.

[33] Smale M, Aguirre A, Bellon M, Mendoza J, Manuel Rosas I (1999)Farmer Management of Maize Diversity in the Central Valleys of Oaxaca, Mexico. CIMMYT Economics Working Paper 99-09 DF: Mexico: International Maize and Wheat Improvement Center.

[34] Bellon MR, Berthaud J, Smale M, Aguirre JA, Taba S et al. (2003) Participatory landrace selection for on-farm conservation: An example from the Central Valleys of Oaxaca, Mexico. Genet Resour Crop Evol 50: 401-416. doi:10.1023/A:1023967611495.

[35] Badstue LB, Bellon MR, Berthaud J, Juárez X, Rosas IM et al. (2006) Examining the role of collective action in an informal seed system: A case study from the Central Valleys of Oaxaca, Mexico. Hum Ecol 34: 249-273. doi:10.1007/s10745-006-9016-2.

[36] Pressoir G, Berthaud J (2004) Patterns of population structure in maize landraces from the Central Valleys of Oaxaca in Mexico. Heredity 92: 88-94. doi:10.1038/sj.hdy.6800387. PubMed: 14666127. 14666127 10.1038/sj.hdy.6800387

[37] Moreno Escobar (2006) Valoracion campesina de la diversidad del maiz: Estudio de caso de dos comunidades indigenas en Oaxaca, Mexico. Ph D Thesis, Universitat Autonoma de Barcelona . 217 p.

[38] Birol E, Villalba ER, Smale M (2008) Famre preferences for milpa diversity and genetically modified maize in Mexico: a latent class approach. Environ Dev Econ 14: 521-540.

[39] Lazos E (2008) La invención de los transgénicos: Nuevas relaciones entre naturaleza y cultura? Nueva Antropol 21: 9-35.

[40] Rice E, Smale M, Blanco JL (1998) Farmers’ use of improved seed selection practices in Mexican Maize: Evidence and issues from the Sierra de Santa Marta. World Dev 26: 1625-1640. doi:10.1016/S0305-750X(98)00079-5.

[41] Latournerie Moreno L, Tuxill J, Moo EY, Arias Reyes L, Cristobal Alejo J et al. (2006) Traditional maize storage methods of Mayan farmers in Yucatán, Mexico: implications for seed selection and crop diversity. Biodivers Conserv 15: 1771-1795. doi:10.1007/s10531-004-6679-0.

[42] Arias LM, Latournerie L, Montiel S, Sauri E (2007) Cambios recientes en la diversidad de maices criollos de Yucatán 23 Mexico: Universidad y Ciência, Tropico Humedo pp. 69-74.

[43] Gil-Munoz A, Lopez PA, Munoz Orozco A, López Sanchez H (2002) Maize (Zea mays L.) Landraces in the state of Puebla, Mexico: Diversity and use. In: Chavez-Servia JL Arias-Reyes LM Jarvis DI Tuxill J Lope-Alzina D . . In: In: Proceedings of a Symposium: Managing Crop Diversity in Traditional Agroecosystems, 13-16 February 2002, Mérida, Mexico: Rome, Italy International Plant Genetic Resources Institute pp. 5-6.

[44] Dyer GA (2005)Smale M Valuing Crop Biodiversity: On-farm Genetic Resources and Economic Change. Wallingford, UK: CAB International pp. 1-16.

[45] Van Dusen ME, Taylor JE (2005) Missing markets and crop diversity: evidence from Mexico. Environ Dev Econ 10: 513–531. doi:10.1017/S1355770X05002317.

[46] Pita Duque A (2010) Impact on maize diversity of Mexican farmers’ participation in off-farm labor markets. Ph D Dissertation, University of California, Davis. 189 p..

[47] Aguirre Gómez JA, Bellon MR, Smale M (2000) A regional analysis of maize biological diversity in Southeastern Guanajuato, Mexico. Econ Bot 54: 60-72. doi:10.1007/BF02866600.

[48] Smale M, Bellon MR, Aguirre Gómez JA (2001) Maize diversity, variety attributes, and farmers’ choices in Southeastern Guanajuato, Mexico. Econ Dev Cult Change 50: 201-225. doi:10.1086/340010.

[49] Chambers KJ, Brush SB, Grote MN, Gepts P (2007) Describing maize (*Zea mays* L.) landrace persistence in the Bajio of Mexico: a survey of 1940s and 1950s collection locations. Econ Bot 61: 60-72. doi:10.1663/0013-0001(2007)61[60:DMZMLL]2.0.CO;2.

[50] Louette D, Charrier A, Berthaud J (1997) In situ conservation of maize in Mexico: Genetic diversity and maize seed management in a traditional community. Econ Bot 51: 20-38. doi:10.1007/BF02910401.

[51] Snively-Martinez AE (2009) Perceptions of change in horticultural subsistence strategies in a rural Mexican community: San Francisco Pichataro, Michoacan. MS Thesis, Washington State University. 158 p..

[52] Rice E (2007) Conservation in a changing world: in situ conservation of the giant maize of Jala. Genet Resour Crop Evol 54: 701-713. doi:10.1007/s10722-006-0023-3.

[53] Van Etten J, de Bruin S (2007) Regional and local maize seed exchange and replacement in the western highlands of Guatemala. Plants Genet Resour 5: 57-70. doi:10.1017/S147926210767230X.

[54] Stromberg PM, Pascual U, Bellon MR (2010) Seed systems and farmers’ seed choices: The case of maize in the Peruvian Amazon. Hum Ecol 38: 539-553. doi:10.1007/s10745-010-9333-3.

[55] Dyer GA, Taylor JE (2008) A crop population perspective on maize seed systems in Mexico. Proc Natl Acad Sci U S A 105: 470-475. doi:10.1073/pnas.0706321105. PubMed: 18184814. 18184814 10.1073/pnas.0706321105 PMC2206560

[56] Dyer GA, Serratos-Hernández JA, Perales HR, Pineyro-Nelson Gepts PA et al. (2009) Escape and dispersal of transgenes through maize seed systems in Mexico. PLOS ONE 4(5): e5734. doi:10.1371/journal.pone.0005734. 19503610 10.1371/journal.pone.0005734 PMC2685455

[57] Sokal RR, Rohlf FJ (1995) Biometry. San Francisco: Freeman. 859pp.

[58] Dyer GA (2002) The cost of in situ conservation of maize landraces in the Sierra Norte de Puebla, Mexico. Ph D Dissertation, University of California, Davis. 168 p..

[59] Dyer G, Taylor JE (2011) The corn price surge: Impacts in rural Mexico. World Dev 39: 1878-1887. doi:10.1016/j.worlddev.2011.04.032.

